# Suicides in the indigenous and non-indigenous populations in the Nenets Autonomous Okrug, Northwestern Russia, and associated socio-demographic characteristics

**DOI:** 10.3402/ijch.v73.24308

**Published:** 2014-05-06

**Authors:** Yury A. Sumarokov, Tormod Brenn, Alexander V. Kudryavtsev, Odd Nilssen

**Affiliations:** 1 Department of Community Medicine, UiT The Arctic University of Norway, Tromsø, Norway; 2 International School of Public Health, Northern State Medical University, Arkhangelsk, Russia

**Keywords:** suicide rates, relative risks, person-years, indigenous Nenets

## Abstract

**Background:**

To describe suicide rates in the indigenous and non-indigenous populations of the Nenets Autonomous Okrug (NAO) in 2002–2012, as well as associated socio-demographic characteristics.

**Study design:**

Retrospective population-based mortality study.

**Methods:**

Data from autopsy reports were used to identify 252 cases of suicide in the NAO in 2002–2012. Data on socio-demographic characteristics of these cases were obtained from passports and medical records at local primary health care units, and were then linked to total population data from the Censuses in 2002 and 2010. Suicide rates for the indigenous Nenets population and the non-indigenous population were standardized to the European standard population. The rates were also estimated according to different socio-demographic characteristics and compared by calculating relative risks.

**Results:**

The crude suicide rates were 79.8 per 100,000 person-years (PYs) in the Nenets population and 49.2 per 100,000 PYs in the non-indigenous population. The corresponding standardized estimates were 72.7 per 100,000 PYs and 50.7 per 100,000 PYs. The highest suicide rates in the Nenets population were observed in the age group 20–29 years (391 per 100,000 PYs), and in females aged 30–39 years (191 per 100,000 PYs). Socio-demographic characteristics associated with high suicide rates in the Nenets population were age 20–39 years, male, urban residence, having secondary school or higher education, being an employee or employer, and being single or divorced. Males aged 20–29 years, and females aged 30–39 and aged 70 years and above had the highest suicide rates in the non-indigenous population (137.5, 21.6 and 29.9 per 100,000 PYs, respectively). The elevated suicide rates observed in the non-indigenous population were associated with male sex, rural residence, secondary school education, being an employee or employer, and being single or divorced.

**Conclusions:**

Suicide rates in the NAO were substantially higher among the indigenous Nenets population than the non-indigenous population, and were associated with different socio-demographic characteristics.

Suicide is one of the most important public health problems among indigenous people worldwide. In the Russian Federation, about 30,000 people commit suicide every year, and probably 10 times as many attempt suicide (1). In Russia, this health problem is much more pronounced in the Far North of the country. Indeed, health workers in the Northern regions, which are also inhabited by many indigenous populations, have reported alarmingly high rates of suicide and destructive lifestyles (2). According to the Russian Federal State Statistics Service, or Rosstat, the Nenets Autonomous Okrug (NAO), a region where indigenous Nenets constitute about one-sixth of the population, has one of the highest suicide rates in Russia (3,4).

High suicide rates in indigenous populations, and the factors associated with this phenomenon, have been addressed by researchers since the 1970s. In 1979, Grove and Lynge (5) showed that suicide rates in the indigenous Inuit population in Greenland increased 4-fold in the 1970s. The authors pointed out several factors, including alcohol consumption, that were associated both with suicide, and the social and cultural evolution of Inuit society.

High suicide rates have also been reported among indigenous Inuit populations from the Hudson Bay (6) and the Canadian Northwest Territories (7). In Northern Norway, there were more suicides among the indigenous Sami population than in the Norwegian general population (8,9). Studies of other indigenous populations, including Aboriginal communities in Australia (10), Native Americans in the United States (11,12) and the Maoris in New Zealand (13), have also shown high suicide rates. In Greenland, Norway (9), Australia (10) and Brazil (14), high suicide risk clusters were found to coincide with the territorial distribution of indigenous populations.

Some common characteristics of suicides among indigenous populations include high suicide rates, especially among young males, and high likelihood of deaths in suicide attempts (8,9). The objectives of this paper are (a) to describe the suicide rates of the indigenous Nenets population and the non-indigenous population in the NAO in 2002–2012 and (b) to define the socio-demographic characteristics associated with suicide in each of these populations.

## Materials and methods

### Study design

The present study is a retrospective population-based mortality study, which included all suicides in the NAO from 1 January 2002 to 31 December 2012.

### Study site and population

**Fig. 1:**
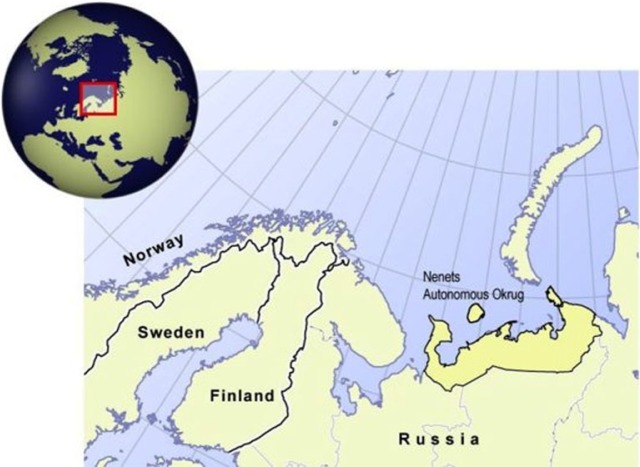
Map of the Nenets Autonomous Okrug (http://www.raipon.org/ikdm/Regions.aspx).

The NAO is situated in the Arctic region of the European part of Russia. The NAO covers about 176.8 thousand square kilometres of Arctic tundra (including the islands Kolguev and Vaigach), which is characterized by very short growing seasons and low temperatures (Fig. 1). According to National Census data, the total population of the NAO was 41,546 in 2002, and 42,090 in 2010 (15,16). The population density in 2012 was 0.24 per square kilometres. The main ethnic groups which make up the population of the NAO are Russians (26,648, 63.3%), Nenets (7,504, 17.8%) and Komi (3,623, 8.6%) (3,15,16).

The Nenets are one of the largest indigenous populations in Russia. They live mostly in 4 Autonomous Okrugs in the Russian Arctic: the NAO, the Yamalo-Nenets Okrug, the Taymyr Okrug and the Khanty-Mansiy Okrug. The Nenets language belongs to Samoedic group of the Ural-Yukagirian language family. Their traditional lifestyle is nomadic or semi-nomadic, and many Nenets live in temporary (seasonal) settlements. The most common occupations are reindeer herding, hunting, fishing and harvesting. The production of handicrafts is also a common occupation of Nenets females (3,15,16).

### Data sources and description

Autopsy reports at the Regional Bureau of Forensic Medicine in the city of Naryan-Mar in the NAO were used to identify cases of suicide in the NAO occurring in 2002–2012. All suicides were examined and autopsied immediately by forensic experts and classified in accordance with the International Classification of Diseases, 10th Revision (codes X60–X84 and Y87.0) (17).

For cases of suicide, data on ethnicity, urban or rural residence, occupation, education level and marital status were obtained from passports and medical records at local primary care units, with support from primary health care workers (doctors, nurses and “feldshers” – rural doctor's assistants), who underwent special training for data collection and registration in December 2010. Correctness of the data obtained from medical records were checked by primary health care workers and double-checked by the first author. Nenets ethnicity was established by forensic experts on the basis of family, passport information, and anthropological findings.

Data for the general population, including population size and population distribution in the NAO by ethnicity and other considered socio-demographic characteristics (sex, urban or rural residence, occupation, education level and marital status), were obtained from the Official Russian Censuses of 2002 and 2010. No other socio-demographic data were available on the NAO population between, or after these 2 Censuses.

### Data analyses

Variables considered in this paper are: age (<10, 10–19, 20–29, 30–39, 40–49, 50–59, 60–69, ≥70 years, and unknown age), ethnicity (indigenous, non-indigenous), sex (male, female), area of residence (urban, rural), employment category (employers and employees; other, including unemployed, retirees, students, dependents and the disabled), education level (university or college, secondary school, incomplete secondary or primary school) and marital status (married, divorced, widowed or single). The variable ethnicity was constructed by allocating the Nenets to the indigenous category, and all others (Russians, Komi, etc.) to the non-indigenous category.

The person-years (PYs) approach was used to estimate suicide rates for the Nenets population and the non-indigenous population, as well as according to the considered socio-demographic characteristics within these populations. The number of PYs of risk in each population was computed as the average population for each ethnicity in the 2002 and 2010 Censuses multiplied by 11 (number of years of observation). Suicide rates were calculated as the number of suicides per 100,000 PYs. For comparability purposes, suicide rates for the Nenets population and the non-indigenous population were standardized to the European standard population by age and sex (18). Relative risk (RR) estimates were calculated as ratios of suicide rates in analogue socio-demographic subgroups of the Nenets population and the non-indigenous population. Microsoft Excel and SPSS 19.0 were used for data storage and analysis.

### Ethical considerations

The study was approved on 23 June 2010 by the Ethical Committee of the Northern State Medical University, Arkhangelsk, Russia.

## Results

Based on autopsy data, 252 suicides were identified in the NAO in 2002–2012, rendering suicide rates of 49.2 per 100,000 PYs in the non-indigenous population and 79.8 per 100,000 PYs in the Nenets population. After standardization to the European standard population, the suicide rate was 50.7 per 100,000 PYs in the non-indigenous population and 72.7 per 100,000 PYs in the Nenets population. Males showed substantially higher suicide rates than females in both the Nenets population and the non-indigenous population (Table I). After stratification by *age* and *sex*, the highest suicide rate was observed among Nenets males aged 20–29 years (391 per 100,000 PYs). Compared to non-indigenous males, Nenets males also showed considerably higher suicide rates in all age groups between 10–19 and 40–49 years. The largest RR value for Nenets males was observed in the 20–29 age stratum (RR=2.84). However, Nenets males aged 50 years and older displayed lower suicide rates than non-indigenous males in the same age groups.

**Table I:** Number of suicides and suicide rates (per 100,000 PYs) by ethnicity, gender and age in the Nenets Autonomous Okrug, 2002–2012

Age (years)	Non-indigenous population			Indigenous (Nenets) population			RR

	Number of suicides	PYs*	Suicide rate	Number of suicides	PYs*	Suicide rate	
Males	161	186,599	86.3	54	39,089	138.1	1.60
<10	0	25,476	0.0	0	8,069	0.0	–
10–19	12	28,600	42.0	6	8,877	67.6	1.61
20–29	45	32,725	137.5	26	6,650	391.0	2.84
30–39	32	30,025	106.6	12	5,638	212.9	2.00
40–49	32	32,032	99.9	7	5,110	137.0	1.37
50–59	25	23,766	105.2	2	2,750	72.7	0.69
60–69	12	9,713	123.5	1	1,397	71.6	0.58
≥70	3	4,208	71.3	0	594	0.0	–
Unknown age	0	55	0.0	0	6	0.0	–
Females	24	189,481	12.7	13	44,831	29.0	2.29
<10	0	23,749	0.0	0	7,755	0.0	–
10–19	3	25,625	11.7	1	8,443	11.8	1.01
20–29	3	29,139	10.3	3	7,194	41.7	4.05
30–39	6	27,715	21.6	7	6,595	106.1	4.90
40–49	6	30,465	19.7	2	6,320	31.6	1.61
50–59	2	25,454	7.9	0	4,125	0.0	–
60–69	0	13,921	0.0	0	2,530	0.0	–
≥70	4	13,371	29.9	0	1,865	0.0	–
Unknown age	0	44	0.0	0	6	0.0	–
Total	185	376,079	49.2	67	83,919	79.8	1.62

* The number of PYs of risk in each group was computed as the mean number of the Census 2002 and Census 2010 multiplied with the number of study years (11 years).

PYs=person-years; RR=relative risk.

The highest suicide rate in Nenets females was observed in the 30–39 age group (106.1 per 100,000 PYs). This same age group also had the largest RR (RR=4.90). There were 6 non-indigenous females 50 years or older who committed suicide, but there were no suicides in the same age group of Nenets females.

The Nenets population with urban residence had substantially higher suicide rates compared to the Nenets population living in rural settings (Table II), and this applied to both males and females. On the contrary, the non-indigenous population with urban residence had lower suicide rates compared to the non-indigenous population with rural residence. Among the non-indigenous population with urban residence, the RR of suicide was 3.14 and was very similar to the corresponding RR in rural settings. Being an employer or an employee was associated with higher suicide rates in both the Nenets population and the non-indigenous population (Table III). The highest suicide rates were observed among those who were employers or employees in both the Nenets males (250.4 per 100,000 PYs) and non-indigenous males (106.7 per 100,000 PYs). The suicide rates in the Nenets population categorized as employer or employee were substantially higher than suicide rates in the same category of the non-indigenous population (RR=2.35 for males, RR=4.45 for females). When the “other” employment category was considered, the risk of suicide in the Nenets population was not substantially different from that in the non-indigenous population.

**Table II:** Number of suicides and suicide rates (per 100,000 PYs) by ethnicity, residence and gender in the Nenets Autonomous Okrug, 2002–2012

Area of residence	Non-indigenous population			Indigenous (Nenets) population			RR

	Number of suicides	PYs*	Suicide rate	Number of suicides	PYs*	Suicide rate	
Urban	132	282,865	46.7	27	18,431	146.5	3.14
Males	112	137,319	81.6	19	7,387	257.2	3.15
Females	20	145,547	13.7	8	11,044	72.4	5.27
Rural	53	93,214	56.9	40	65,489	61.1	1.07
Males	49	49,280	99.4	35	31,702	110.4	1.11
Females	4	43,934	9.1	5	33,787	14.8	1.63

* The number of PYs of risk in each group was computed as the mean number of the Census 2002 and Census 2010 multiplied with the number of study years (11 years).

PYs = person-years; RR = relative risk.

**Table III:** Number of suicides and suicide rates (per 100,000 PYs) by ethnicity, employment status and gender in the Nenets Autonomous Okrug, 2002–2012

	Non-indigenous population			Indigenous (Nenets) population			RR

	Number of suicides	PYs*	Suicide rate	Number of suicides	PYs*	Suicide rate	
Employers and employees	118	195,657	60.3	42	29,414	142.8	2.37
Males	106	99,314	106.7	33	13,178	250.4	2.35
Females	12	96,344	12.5	9	16,236	55.4	4.45
Other**	67	176,556	37.9	25	54,456	45.9	1.21
Males	55	85,404	64.4	21	25,883	81.1	1.26
Females	12	91,152	13.2	4	28,573	14.0	1.06

* The number of PYs of risk in each group was computed as the mean number of the Census 2002 and Census 2010 multiplied with the number of study years (11 years).

** Includes unemployed, retirees, students, dependents and the disabled.

PYs = person-years; RR = relative risk.

*Education level* showed an association with the risk of suicide in both the Nenets population and the non-indigenous population (Table IV). The highest rate was found in Nenets males with secondary school education (500.1). Among those who had completed secondary school, the higher suicide rate was observed in the Nenets population (RR=2.73 for males and 3.34 for females). In the strata of incomplete secondary or primary education, Nenets males showed substantially higher suicide rates compared to non-indigenous males (RR=4.57).

**Table IV:** Number of suicides and suicide rates (per 100,000 PYs) in the study sample aged of 10 years and older by ethnicity, education and gender in the Nenets Autonomous Okrug, 2002–2012

Education level	Non-indigenous population			Indigenous (Nenets) population			RR

	Number of suicides	PYs*	Suicide rate	Number of suicides	PYs*	Suicide rate	
University/college	21	44,930	46.7	4	3,064	130.6	2.79
Males	19	18,486	102.8	3	710	422.8	4.11
Females	2	26,444	7.6	1	2,354	42.5	5.62
Secondary school	148	151,481	97.7	44	20,884	210.7	2.16
Males	131	71,418	183.4	34	6,798	500.1	2.73
Females	17	80,064	21.2	10	14,086	71.0	3.34
Incomplete secondary or primary school	16	126,841	12.6	19	44,116	43.1	3.41
Males	11	69,482	15.8	17	23,502	72.3	4.57
Females	5	57,360	8.7	2	20,614	9.7	1.11

* The number of PYs of risk in each group was computed as the mean number of the Census 2002 and Census 2010 multiplied with the number of study years (11 years).

PYs = person-years; RR = relative risk.

Fifty suicides (5 Nenets and 45 non-indigenous) were excluded from Table IV because of missing data on marital status. In both ethnicities, the highest suicide rates were observed among single males (Table V). There were no suicides among widows and widowers in the Nenets population. In all the other categories of marital status, suicide rates were higher in the Nenets population than the non-indigenous population.

**Table V:** Number of suicides and suicide rates (per 100,000 PYs) in the study sample aged 16 years and older by ethnicity, marital status and gender in Nenets Autonomous Okrug, 2002–2012

	Non-indigenous population			Indigenous (Nenets) population			RR

	Number of suicides	PYs*	Suicide rate	Number of suicides	PYs*	Suicide rate	
Married	74	175,951	42.1	29	26,763	108.4	2.58
Males	68	91,102	74.6	20	10,907	183.4	2.46
Females	6	84,849	7.1	9	15,857	56.8	8.03
Single	45	64,427	69.8	30	22,391	134.0	1.92
Males	38	38,291	99.2	26	12,771	203.6	2.05
Females	7	26,136	26.8	4	9,620	41.6	1.55
Divorced	11	24,387	45.1	2	1,892	105.7	2.34
Males	11	9,416	116.8	2	627	319.0	2.73
Females	0	14,971	0.0	0	1,265	0.0	–
Widow/widower	8	25,377	31.5	0	5,841	0.0	–
Males	6	3,350	179.1	0	946	0.0	–
Females	2	22,028	9.1	0	4,895	0.0	–

* The number of PYs of risk in each group was computed as the mean number of the Census 2002 and Census 2010 multiplied with the number of study years (11 years).

PYs = person-years; RR = relative risk.

## Discussion

This study shows a substantially higher suicide rate among the indigenous Nenets population when compared with the non-indigenous population in the NAO.

Males showed higher suicide rates than females in both the Nenets and the non-indigenous populations. This sex effect is in accordance with a report by Durkheim (19). In the present study, males aged 20–29 years in both the Nenets population and the non-indigenous population had the highest risk of suicide. This is probably the most vulnerable age for males in relation to suicide, as it is a time when they graduate from school and begin their military service. They may not get the job they want, they may be lost between fantasy and reality and may enter into sensitive romantic relationships. Moreover, males in this age group are more exposed to alcohol, because they are past the legal drinking age of 18 years. Nenets females had the highest risks of suicide in the age group 30–39 years. The critical age period for suicide may be later for females because of factors such as family and marriage; as having small children and family obligations may postpone suicidal ideations (20). The defined age and sex associations we found for suicide in the NAO are in line with findings of other studies performed in Russia (1) and abroad (21,22). Relatively low suicide rates in Nenets males over 50 may be explained by the short life expectancy of Nenets males, which was below 56 years during the study period (15,16).

Nenets living in urban areas had higher suicide rates than non-indigenous populations. This is contrary to what has been seen in other areas of Russia, where rural populations have higher suicide rates than urban populations (20,23). One reason for this maybe a lack of integration of the traditional lifestyle, accompanied by easier access to alcohol in urban areas. Indeed, the suicide-protective effect of traditional lifestyle and strong sense of belonging has been reported in studies of Aboriginal populations (2,4,24).

The non-indigenous population living in rural areas showed high suicide rates. This is in line with the situation in most territories of Russia (20). For example, individuals living in a rural area of the neighbouring Arkhangelsk region had higher suicide rates than those living in urban areas of the same region (25). Social deprivation is more common among those living in rural than urban areas of Russia, especially among non-indigenous populations. Economic depression, unemployment, heavy alcohol consumption, etc. are also more prevalent in rural areas (4,24). Similar findings have been reported in other studies (26).

Losing a job and long-term unemployment are well-known factors associated with suicide (27). However, our study showed a higher RR among Nenets males and females who were either employers or employees. A smaller but still increased risk was also observed among non-indigenous males who were either employers or employees compared to non-indigenous males in the “other” category of employment. Similar findings were reported in Asian studies where suicide rates in the employed were higher than in unemployed individuals (28).

Nenets with a university education had higher suicide rates compared to the non-indigenous population with the same education level. One possible explanation is that the Nenets are far removed from their traditional nomadic lifestyle when they reside in an urban area. Once they get a higher education, they normally leave their society of origin, and move to large cities in Russia – away from their culture and traditions. Although they get away from their own culture, they may not integrate Russian culture well either, and as a result may become rootless and alone. This also corresponds well with Durkheim's theory (19). Higher education has been associated with a high risk of suicide in several other studies (29,30).

When considering the interconnection of employment, education level and urban residence, one can see that all 3 were associated with suicide in the Nenets population. This triad seems to act as a combined factor that is associated with a lack of “sense of indigenous belonging,” lack of cultural identity and problems of resilience (31).

Divorced males (Nenets and non-indigenous) and married Nenets females showed the highest risks of suicide. Divorced females had no suicides. Several researchers (32,33) have suggested that divorce might be a protective factor for suicide, especially for females. Divorce dramatically changes the social and sometimes the economic life of males because of financial and other losses. For females divorce is less financially dangerous but often seems to act as an escape from domestic violence and social deprivation encountered in marriage (32). A few studies have shown that being single (widowed, divorced or unmarried) increases the risk of suicide (32,34,35). However, there is also evidence that it decreases the risk of suicide in women (36).

### Limitations

Socio-demographic data for the general population were obtained only from the 2002 and 2010 Censuses. There were no other available data on age and sex distribution, residence, education, occupation and marital status for the population of the NAO during the study period. For these reasons, the denominators in our calculations of suicide rates were estimates rather than true numbers. This may have biased our results to some degree, but this potential bias cannot be sufficiently large to explain our findings.

No information on history of self-harm or suicide attempt was available for the cases of suicide included in the present study, and marital status was not defined in 50 (19.8%) cases. Census data consisted of the different subgroups of jobless people including pensioners and those who were getting the different types of financial aid from the state. The proportion of officially “unemployed” (registered in the database of labour services) in the NAO is very low (3). Otherwise we have tried to minimize the limitations by performing a detailed analysis of each case included in the database.

### Strengths

The comprehensive information collected by forensic experts in autopsy reports may be seen as strength in our study. Indeed, this work was well organized in the NAO, with planes and helicopters used when necessary to transport forensic experts to the sites of possible suicides. The region is relatively transparent and it is easy to get a good overview. Moreover, we had the cooperation of all local primary health care centres and rural health care workers for the present study.

### Validity and completeness of data

According to Russian legislation, all suicides in Russia are investigated by the police and the Investigation Committee of Russian Federation in order to identify potential criminal cases. A forensic examination immediately follows the primary police investigation. The key role of the forensic experts is to define the cause of death and according to the International Classification of Diseases, 10th Revision (1,17).

## Conclusions

The overview of suicide in the NAO was characterized by higher suicide rates in the indigenous Nenets population compared with the non-indigenous population. The present study showed that suicides among Nenets and non-indigenous populations in the NAO are associated with different socio-demographic characteristics. The strongest positive associations with the suicidal risk in the Nenets population were observed for age 20–29 years, male, urban residence and high education level for both sexes, being divorced or a widower for males, and being married for females. These characteristics may have connections to a lack of a “sense of indigenous belonging,” lack of cultural identity and problems of resilience.

In the non-indigenous population, higher risks of suicide were observed for males, rural residence, having secondary school education, being an employer or employee and being single. The highest suicides rates in this group were seen in males aged 20–29 years, and females aged 30–39 and 70 years and above. This information could be used to create targeted prevention activities in different population groups in the Arctic.
